# PolyCat: A Resource for Genome Categorization of Sequencing Reads From Allopolyploid Organisms

**DOI:** 10.1534/g3.112.005298

**Published:** 2013-03-01

**Authors:** Justin T. Page, Alan R. Gingle, Joshua A. Udall

**Affiliations:** * Plant and Wildlife Science Department, Brigham Young University, Provo, Utah 84062; † Plant Genome Mapping Laboratory, University of Georgia, Athens, Georgia, 30602

**Keywords:** read mapping, polyploid, cotton, SNP, homoeo-SNP

## Abstract

Read mapping is a fundamental part of next-generation genomic research but is complicated by genome duplication in many plants. Categorizing DNA sequence reads into their respective genomes enables current methods to analyze polyploid genomes as if they were diploid. We present PolyCat—a pipeline for mapping and categorizing all types of next-generation sequence data produced from allopolyploid organisms. PolyCat uses GSNAP’s single-nucleotide polymorphism (SNP)-tolerant mapping to minimize the mapping efficiency bias caused by SNPs between genomes. PolyCat then uses SNPs between genomes to categorize reads according to their respective genomes. Bisulfite-treated reads have a significant reduction in nucleotide complexity because nucleotide conversion events are confounded with transition substitutions. PolyCat includes special provisions to properly handle bisulfite-treated data. We demonstrate the functionality of PolyCat on allotetraploid cotton, *Gossypium hirsutum*, and create a functional SNP index for efficiently mapping sequence reads to the D-genome sequence of *G. raimondii*. PolyCat is appropriate for all allopolyploids and all types of next-generation genome analysis, including differential expression (RNA sequencing), differential methylation (bisulfite sequencing), differential DNA-protein binding (chromatin immunoprecipitation sequencing), and population diversity.

Read-mapping is a fundamental part of next-generation genomic research. Read-mapping was the essential first-step in pioneering studies of gene expression (Mortazavi *et al.* 2008; Wang *et al.* 2009), quantification of genome methylation (Lister *et al.* 2008; 2009), estimation of DNA−protein interactions (Park 2009; Wilbanks and Facciotti 2010), and assessment of population diversity (Sabeti *et al.* 2007; Durbin *et al.* 2010; Chia *et al.* 2012). Researchers have largely applied these methodologies to diploid genomes of model organisms, including *Arabidopsis thaliana* (Zhang *et al.* 2006; Vaughn *et al.* 2007; Cokus *et al.* 2008; Lister *et al.* 2008; Kaufmann *et al.* 2010), *Drosophila melanogaster* (Graveley *et al.* 2010; McManus *et al.* 2010; Nègre *et al.* 2011), and *Homo sapiens* (Mortazavi *et al.* 2008; Valouev *et al.* 2008; Lister *et al.* 2009; Trapnell *et al.* 2010).

Read-mapping will also be used to analyze the polyploid genomes of many important plants. It has been recently established that all seed plants are paleopolyploids, with all angiosperms sharing an additional event (Jiao *et al.* 2011). Thus, all flowering plants have undergone at least two paleopolyploid events in its history. Although all flowering plants have a history of whole-genome duplication (Stebbins 1950; Adams and Wendel 2005; Paterson *et al.* 2005; Cui 2006; Wood *et al.* 2009; 2011), ancient duplications do not significantly complicate read-mapping because duplicated loci diverge over time, permitting confident placement of a large majority of sequencing reads. On the other hand, more recent whole-genome duplications challenge read mapping by causing a twofold increase in chromosome number and DNA sequence while preserving gene order, coding and noncoding sequence, and chromosomal elements such as centromeres and telomeres. The increasing capacity of DNA sequencing will allow future studies to address the evolutionary and molecular hypothesis of recent polyploidization events (Osborn *et al.* 2003; Adams and Wendel 2005; de Peer *et al.* 2009; Flagel and Wendel 2009) and the effects of polyploidization on plant phenotypes (Gaeta and Pires 2010; Soltis *et al.* 2004; Schranz 2000; Dubcovsky and Dvorak 2007). Accurate assignment of sequencing reads to their genomes-of-origin will be essential to elucidate the underlying principles and consequences of polyploid evolution.

Because most read-mapping software has been written for the analysis of diploid genomes (Griffith *et al.* 2010; Wu and Nacu 2010; Garber *et al.* 2011; Langmead and Salzberg 2012), they are unsuited for mapping sequencing reads from polyploid samples for two reasons. First, mapping reads from a polyploid to a related diploid genome results in differential mapping efficiencies because one coresident genome matches the reference better than the other. Differential mapping efficiency biases subsequent comparisons of the two genomes and skews quantitative analyses. Second, existing tools cannot distinguish between the two genomes to assign quantitative results to one or the other. Other phenomena, such as copy number variation, cause different problems for interpreting read mapping results and are not the focus of this effort (Kitzman *et al.* 2012).

The problems related to analysis of polyploid data can be mitigated by *a priori* single-nucleotide polymorphism (SNP) identification within and between extant diploid relatives. Most of these SNPs are vertically inherited from diploid ancestors to allopolyploid derivatives, so they are present both between diploid relatives and between coresident homeologous genomes of the allopolyploid. These “homoeo-SNPs” can be used to reduce mapping efficiency bias through the use of SNP-tolerant mapping, as with heterozygous genes in humans (Wu and Nacu 2010). After mapping, the genome of origin for individual reads can be identified based on a comparison between the bases at the homoeo-SNP locus and the respective bases of related diploid species—a process we call read categorization.

Bisulfite-treated data present additional challenges to read mapping and read categorization because transition SNPs cannot be distinguished from bisulfite (BS) conversion events. Because transition SNPs comprise a majority of all SNPs, including homoeo-SNPs, treatment with BS causes a majority of homoeo-SNPs to be potentially uninformative for categorizing BS sequencing (BS-seq) reads.

Here we present PolyCat: a pipeline for mapping and categorizing sequencing reads from allopolyploid genomes. PolyCat was developed and tested on data derived from various species of cotton (genus *Gossypium*). The most common form of domesticated cotton (*Gossypium hirsutum*) is an allopolyploid composed of homeologous A_T_- and D_T_-genomes, where the ‘T’ subscript indicates genomes within the tetraploid nucleus (Wendel and Cronn 2003). Two extant diploid cotton species have genomes closely related to those contained in the polyploid nucleus, namely the A_2_-genome of *G. aboreum* and the D_5_-genome of *Gossypium raimondii*. The A_2_-genome is more closely related to the A_T_-genome than the D_5_ genome to the D_T_-genome (Senchina 2003; Flagel *et al.* 2012); however, the diploid D_5_-genome recently was sequenced because of its smaller size (Paterson *et al.* 2012). This characterized trio of genomes was used to develop and evaluate the read mapping and read categorization of PolyCat.

The PolyCat source code and the current cotton SNP-index is publically available for other studies (http://cottonevolution.info), along with a web portal in which evaluation sequence data sets may be submitted for mapping and categorizing. PolyCat produces genome-specific BAM files as output, which may be immediately used by most current bioinformatics tools for downstream analyses, such as differential expression (RNA-sequencing [RNA-seq]), differential methylation (BS-seq), differential DNA-protein binding (chromatin immunoprecipitation sequencing), and population diversity.

## Materials and Methods

### Sequence preprocessing and SNP index generation from diploid-derived data

Sickle (https://github.com/najoshi/sickle) was used to trim all sequence reads with a quality cutoff of 20. We used the Genomic Short-read Nucleotide Alignment Program (GSNAP) (Wu and Nacu 2010) to map 1,140,550,335 reads from *G. raimondii* (D_5_), and 4,070,680,434 reads from *G. arboreum* (A_2_) to the *G. raimondii* reference genome (Paterson *et al.* 2012), accepting only unique best hits and allowing for novel splice sites (Table 1). SAMtools (Li *et al.* 2009) was used to generate two pileups, one for A_2_ and one for D_5_. We compared the resulting pileups with each other and with the D_5_ reference at each nucleotide position to identify homoeo-SNPs between the genomes, as well as allelic SNPs within the A_2_ and D_5_ genomes with at least 4× coverage and a minor allele frequency of 40%. Sequences used in this effort are available through the National Center for Biotechnology Information Sequence Read Archive (Table 1).

**Table 1:** Contribution of different DNA and RNA sources to construction of a SNP index

Sequence Source	A_2_	D_5_	SRA IDs
ISU fiber, leaf, buds, floral parts, seed (RNA-seq)	1,032,531,096	931,721,308	SRA061240 SRA061456
BYU Petal RNA-seq	42,047,506	39,974,015	SRA062614
Whole G. Shotgun (Genomic DNA)	2,996,073,656	168,243,740	SRA062615
Total	4,070,652,258	1,139,939,063	

SNP, single-nucleotide polymorphism; SRA, Sequence Read Archive (National Center for Biotechnology Information).

### RNA-seq read categorization

**Figure 1:**
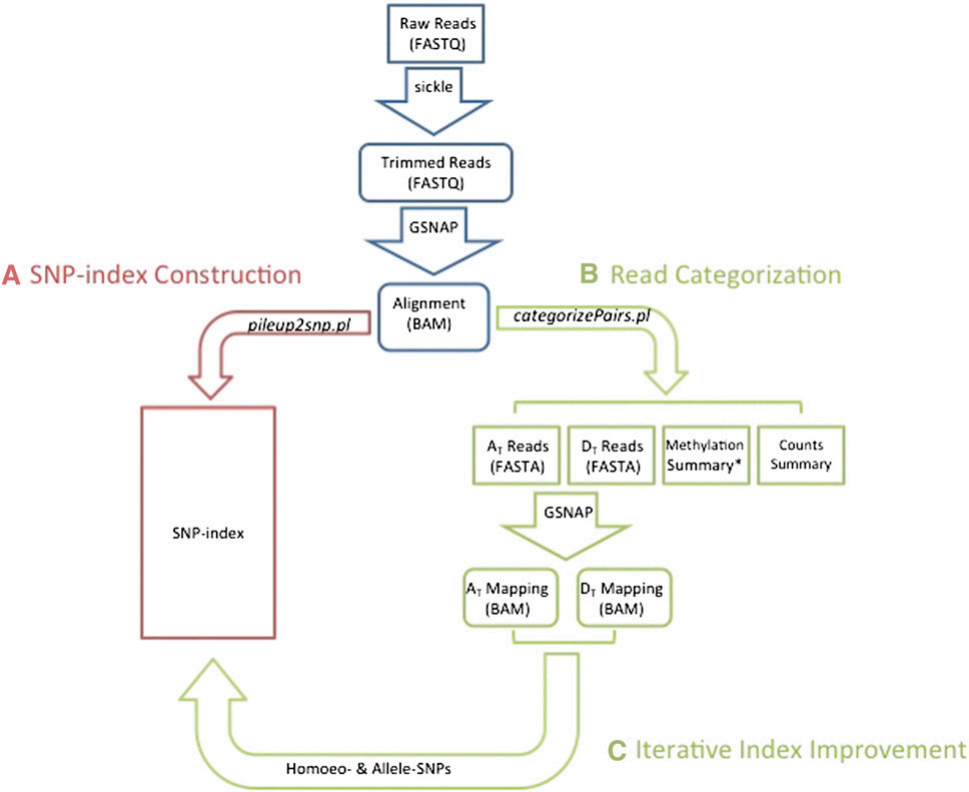
A diagram of the PolyCat read categorization process. (A) Reads from diploids are used to generate an index of homoeo-SNPs. (B) Reads from tetraploids are assigned to a genome based on the sequenced base at each overlapped SNP position. (C) Categorized reads from tetraploids can then be realigned into genome-specific assemblies and used to improve the SNP-index.

We illustrate read categorization with RNA-seq reads from cotton petals in two allopolyploid cotton species: *G. hirsutum* (cv. Maxxa Acala and referred to as Maxxa) and *Gossypium tomentosum*, an endemic polyploid cotton species of Hawaii. Because the cotton A_T_ and D_T_ genomes are more similar to their extant diploid relatives than they are to each other (Flagel *et al.* 2012), SNPs between diploids approximated SNPs between their respective allopolyploid genomes and were considered putative homoeo-SNPs. These SNPs were used to categorize reads from *G. hirsutum* and *G. tomentosum* as originating from either the A_T_ or D_T_ genomes (Udall 2006a,b; Yang *et al.* 2006; Byers *et al.* 2012; Flagel *et al.* 2012). After mapping to the D_5_-genome reference as described previously, PolyCat was used to compare the nucleotide at each SNP position to the SNP index and categorized it as A_T_-genome or D_T_-genome (Figure 1), depending on its unique match in the SNP index. PolyCat then assigned each read to a category based on the number of A_T_-genome and D_T_-genome matches. Reads with at least 75% (a user-specified parameter) of matches for one genome were categorized as A_T_ or D_T_, accordingly. Reads with matches to both were categorized as chimeric (X). Reads without SNP positions or matches were categorized as unknown (N).

### Bisulfite sequencing

Bisulfite treatment deaminates unmethylated cytosines to uracils. During subsequent polymerase chain reaction, the uracil is interpreted as a thymine for complementary strand synthesis. After sequencing, cytosine-to-thymine mismatches (C→T) between the read and the reference sequence indicate unmethylated cytosines on the sequenced ‘+’ strand. Guanine to arginine mismatches (G→A) indicate unmethylated cytosines on the sequenced ‘−’ strand. This conversion looks like a transition SNP and requires tracking by PolyCat to avoid data loss.

**Figure 2:**
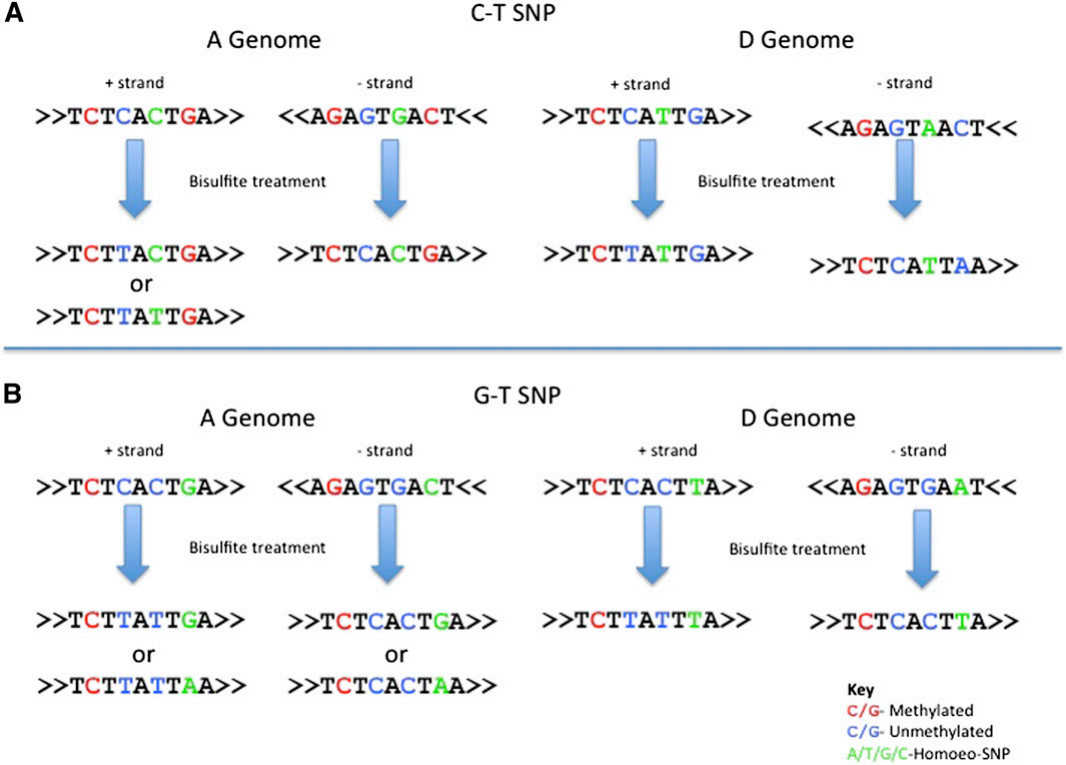
Homoeo-SNPs in BS treatment. (A) Suppose there is a C-T SNP on the ‘+’ strand between the A and D genome (green characters). After BS treatment, reads ‘descending’ from the ‘+’ strand may have a C or a T, depending on the methylation state. All reads from the ‘−’ strand will have a ‘C’ at that SNP position, regardless of methylation state. And in this case, all reads from the D genome will have a T, regardless of the strand. Thus, a T base at the SNP is uninformative because it could be from the D genome or an unmethylated A genome. However, if it were known that the T nucleotide was descended from the ‘−’ strand, then the T would be fully informative (*i.e.*, it would indicate the read was unambiguously from the D-genome in this example). As mentioned in *Materials and Methods*, we impute the original read strandedness based of the frequency C→T and G→A transitions. (B) Suppose there is a G→T SNP; there is no ambiguity, then, about the genome origin of the original strand because A-genome reads will have a G or an A, whereas D-genome reads will have a T.

For BS-treated data, PolyCat first inferred the origin strand of each read by counting C→T and G→A conversions. More C→T conversions indicated ‘+’ strand, whereas more G→A conversions indicated ‘−’ strand. Ambiguous strands were counted as half reads for both strands. For ‘+’ strand reads, PolyCat accepted a thymine as a match for a cytosine allele; for ‘−’ strand reads, PolyCat accepted an adenosine as a match for a guanine. Knowing the strand origin allowed PolyCat to maximize information from each SNP. Because transition SNPs comprised the majority of the SNP index (Table 2), most reads would be uncategorizable if transition SNPs were made uninformative. However, C-T SNPs were uninformative only on the ‘+’ strand, and G-A SNPs only on the ‘−’ strand (Figure 2). So PolyCat could use C-T SNPs to categorize ‘−’ strand reads and G-A SNPs to categorize ‘+’ strand reads to minimize data loss.

**Table 2:** Composition of SNP index by SNP type

At-genome	Dt-genome			
	A	T	C	G
A	0	190,935	132,443	409,059
T	190,468	0	407,605	132,678
C	117,349	363,240	0	86,903
G	363,609	117,194	87,509	0

After categorizing each read, PolyCat reported the number of cytosines and thymines, or guanines and adenosines, at each cytosine or guanine reference position, along with the methylation context—CG, CHG, or CHH—according to the D_5_-genome reference (Lister and Ecker 2009). Separate columns reported the total number of cytosines and thymines, as well as the counts for each genome (A_T_, D_T_, X, or N).

## Results

### Homeologous SNP index

A SNP index largely composed of differences between homeologous loci was created by comparing the alignments of reads from A- and D-genome diploid species (A_2_ and D_5_, respectively) to the D_5_-genome reference. We refer to these single-nucleotide differences between homeologous loci as homoeo-SNPs. Our SNP index consisted of 2,633,689 SNPs (Table 2). Of these, 1,543,513 (~58.6%) were transitions (tr) and 1,055,479 were transversions (tv), a ratio of approximately 1.5 (34,697 SNPs had multiple allele possibilities in one of the two genomes and could not be classified). The gene-dense Maize HapMap 1 had a tr/tv ratio of approximately 1.0 (Gore *et al.* 2009), and the more uniform Maize HapMap 2 has a tr/tv ratio of approximately 2.0 (Chia *et al.* 2012), demonstrating a greater abundance of transition SNPs in intergenic regions in which natural selection does not prevent spontaneous cytosine to thymine mutations (Coulondre *et al.* 1978). These values, together with the cotton SNP-index tr/tv ratio of 1.5, suggest a correlation between the genic skew of a SNP collection and the tr/tv ratio (Supporting Information, Table S1).

SNPs were distributed evenly across the genome, reflecting the gene density of the *G. raimondii* genome. The average SNP density across all chromosomes was approximately 3.51 SNPs/kbp (Table S2). Chromosomes 6, 7, and 9 had slightly more than 4 SNPs/kbp, whereas Chromosomes 5, 10, and 13 had slightly less than 3 SNPs/kbp. The remaining chromosomes had between 3 and 4 SNPs/kbp.

**Figure 3:**
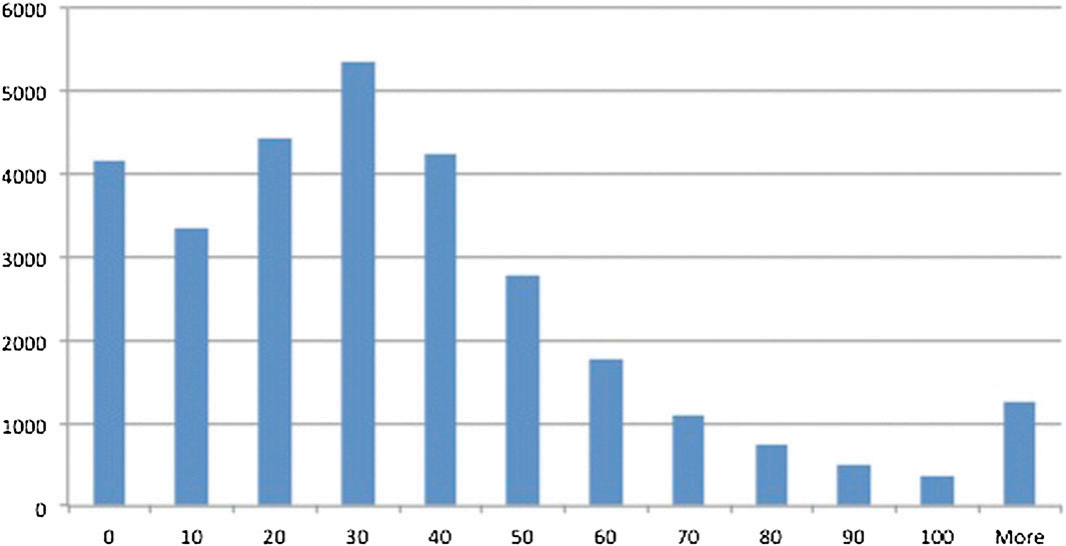
Histogram of SNP frequencies by gene as annotated in the initial draft of the D-genome reference sequence. Most genes (mode) had between 20 and 30 SNPs. A total of 7235 genes with low coverage (RNA-seq or WGS) from the diploid datasets were removed from the distribution.

A total of 1,123,129 SNPs were in annotated genes, including 579,259 in exonic sequence (9.4 SNPs/kbp). This increased SNP density in genes was likely due to increased sequence conservation between the A- and D-genomes. (Cronn *et al.* 2002; Senchina 2003). The number of SNPs varied greatly between genes (Figure 3). A binomial distribution of genes with 9.4 SNPs/kbp and 1.6 kbp of average length predicted 0 genes with no coding homoeo-SNPs, but 4161 genes actually had no coding homoeo-SNPs. These data suggest strong purifying selection on these genes, possibly due to their connectedness (Birchler *et al.* 2005; Freeling and Thomas 2006).

### SNP-tolerant mapping efficiency

SNPs between diploid relatives can approximate homoeo-SNPs between coresident genomes of an allopolyploid (Bancroft *et al.* 2011; Harper *et al.* 2012; Lai *et al.* 2012). SNP-tolerant mapping uses these SNPs to improve mapping efficiencies of sequence reads from allopolyploid genomes, but previous efforts (*e.g.*, *Brassica napus* and *Tuber aestivum*) have not used SNP-tolerant mapping. To demonstrate the effectiveness of SNP-tolerant mapping, GSNAP (Wu and Nacu 2010) was used to map sequence reads from A_2_, D_5_, Maxxa, and a synthetic F_1_ hybrid to the D_5_ reference. The mappings were performed with and without SNP-tolerant mapping. For comparison, Bowtie also was used to map the WGS reads from A_2_ and D_5_ to the D_5_ reference (Langmead *et al.* 2009).

**Figure 4:**
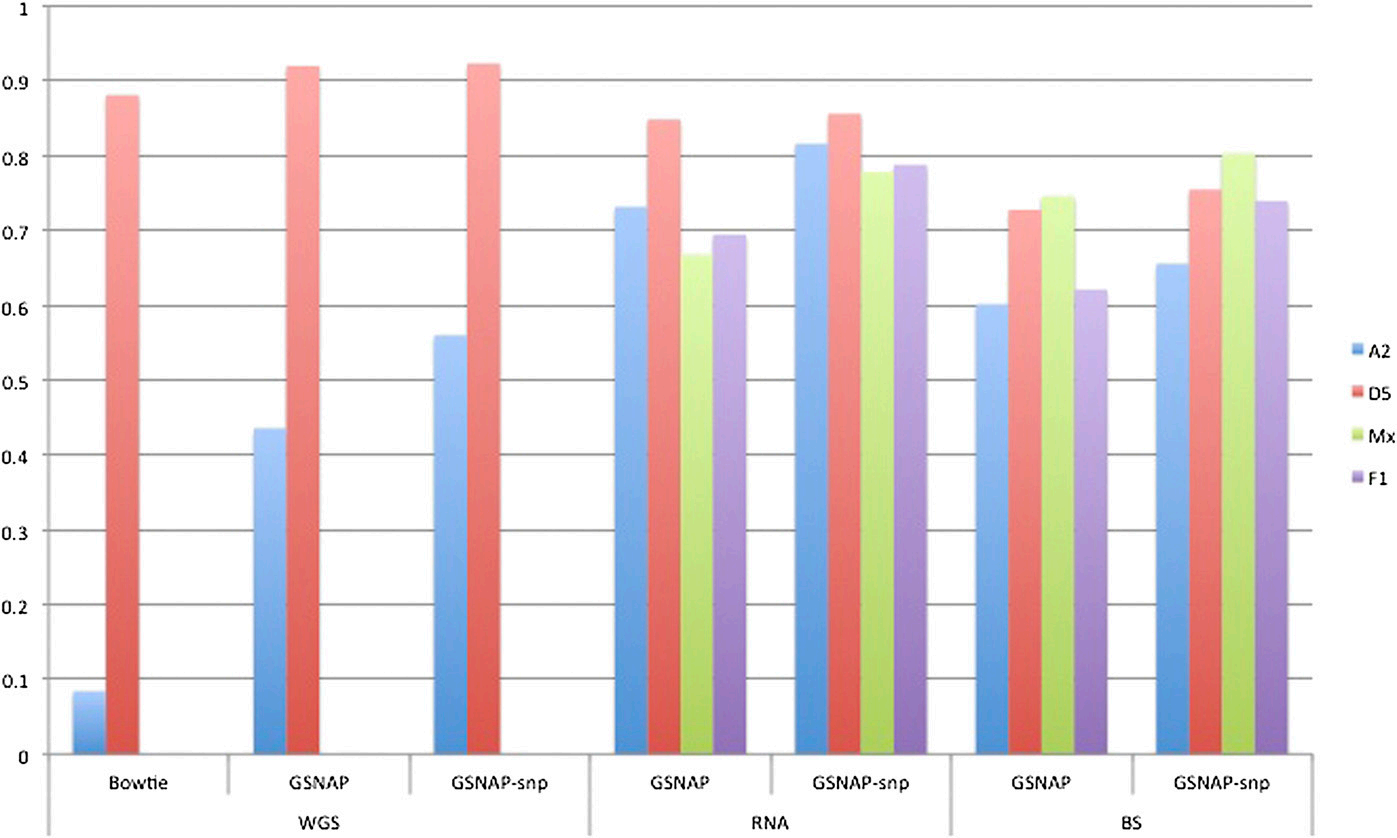
Mapping efficiency with and without SNP-tolerant mapping. Reads were mapped by Bowtie (WGS only), GSNAP, and GSNAP with SNP-tolerant mapping (GSNAP-snp). WGS reads from *G. arboreum* (A2), *G. raimondii* (D5), were mapped to the reference genome of *G. raimondii*. Subsequently, RNA-seq and BS-seq reads from A2, D5, *G. hirsutum* (Mx) and the F1 diploid hybrid (F1) also mapped using SNP-tolerant mapping.

The SNP-tolerant mapping substantially improved the mapping efficiency of reads from A_2_ or allopolyploid cotton to the D_5_-genome reference (Figure 4). The mapping efficiency of D_5_ reads to the D-genome reference was unchanged. GSNAP mapped more A_2_ reads than Bowtie, and a substantial increase of mapping efficiency was observed with SNP-tolerant mapping enabled. Of that increase, approximately 50% was observed whereas mapping A_2_ BS-treated reads because of the reduced sequence complexity typical of BS treatment (Lister and Ecker 2009; Laird 2010; Krueger *et al.* 2012). The overall mapping efficiency also improved for the allopolyploid reads ince allopolyploid reads included both A_T_-genome and D_T_-genome reads. The improved efficiency of allopolyploid cotton reads was a result of accurate mapping of A-genome reads to the diploid D-genome reference.

### Read categorization of sequence reads

**Figure 5:**
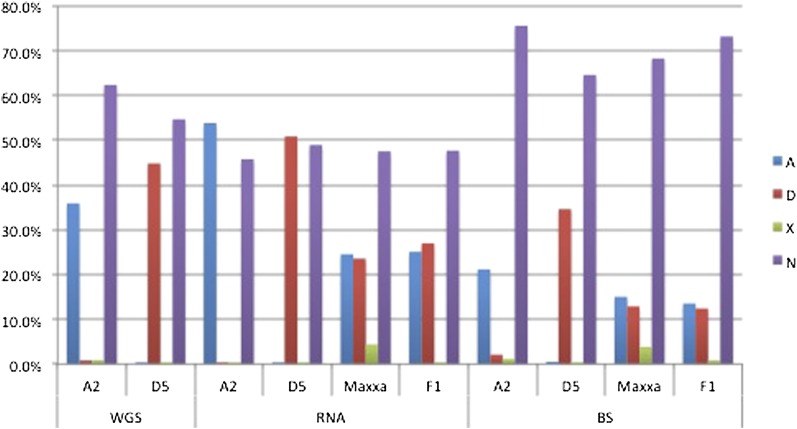
Percentages of read categorization. Reads were mapped to the *G. raimondii* reference with GSNAP and SNP-tolerant mapping, then categorized as A-genome (A), D-genome (D), chimeric (X), or unknown (N).

After mapping, PolyCat categorized each read based on matches to the SNP index (Figure 5). To test accuracy, reads from diploids were also categorized. Most reads were assigned to their correct genome (0.3% of D_5_ reads categorized as A_T_ and 0.8% of A2 reads categorized as D_T_). Erroneous categorization occurred most frequently in BS-treated reads (2.1%). A small number of reads from diploids (<1%) were categorized as chimeric, indicating nucleotide matches at separate loci (within a read) to both the A- and D-genomes. Chimeric reads were slightly more common in A_2_ than D_5_. The low level of erroneous or chimeric categorization shows that PolyCat successfully categorized the vast majority of sequence reads.

For allopolyploid reads, erroneous categorization was impossible to definitively identify, but the rate of chimeric categorization was low, albeit greater than in reads from diploids (4.4% in RNA-seq and 3.8% in BS-treated reads). Two factors may explain the increase in chimeric categorization in reads from allopolyploids: (1) The SNP index was based on A_2_ and D_5_, so it includes false homoeo-SNPs that are really allelic SNPs specific to A_2_ or D_5_. (2) After polyploidization, gene (or intergene) conversion events between the allopolyploid genomes could have replaced the nucleotides of one genome with the nucleotides of the other. At homoeo-SNP positions, conversion events can be detected in reads from an allopolyploid (Salmon *et al.* 2009; Flagel *et al.* 2012), and the rate of nonreciprocal homeologous exchange had been extrapolated to be approximately 2% between the two genomes (Salmon *et al.* 2009). A greater rate of nonreciprocal homeologous exchange (6.8%) was recently detected in a global assembly of expressed sequence tags from *G. hirsutum* and *G. barbadense* (Flagel *et al.* 2012). If homeologous exchanges did not overlap a homoeo-SNP position or if they were larger than individual read (or expressed sequence tags), then they were not detected. Thus, these numbers likely underestimate the true number of historical exchanges between the two genomes.

Approximately one-half of the polyploid reads could not be categorized because they did not overlap a homoeo-SNP. The uncategorized fraction of reads varied by length and by quality of reads. In the reference genome, only 163 Mb of 749 Mbp were within 100 bp (the length of Illumina HiSeq reads in our dataset) of a homoeo-SNP, resulting in a 21.78% theoretical probability of any whole genome shotgun read being categorized. Genic regions (120 Mbp) had a greater density of putative homoeo-SNPs than intergenic regions because of our large collection of diploid RNA-seq data. In these regions, the theoretical probability of categorization was higher (60.7%) than the remainder of the genome (Figure 5). These data illustrate the dependency of polyploid reads categorization on SNP density.

The BS-treated reads had a decreased level of uncategorized reads because of the information loss caused by BS conversion. Each transition homoeo-SNP was only informative for half of the reads (C-T SNPs for ‘+’ strand reads and A-G SNPs for ‘−’ strand reads). Although the same portion of the genome (120 Mbp) could have been theoretically be categorized after BS treatment, the combination of transitions confounded with BS treatment and of uneven distribution of homoeo-SNP density (*e.g.*, < single homoeo-SNP/read) caused fewer reads to be categorized in some regions than would have been otherwise categorized had only one of the individual causes been a factor.

### Allele-SNPs within individual allopolyploid genomes

**Figure 6:**
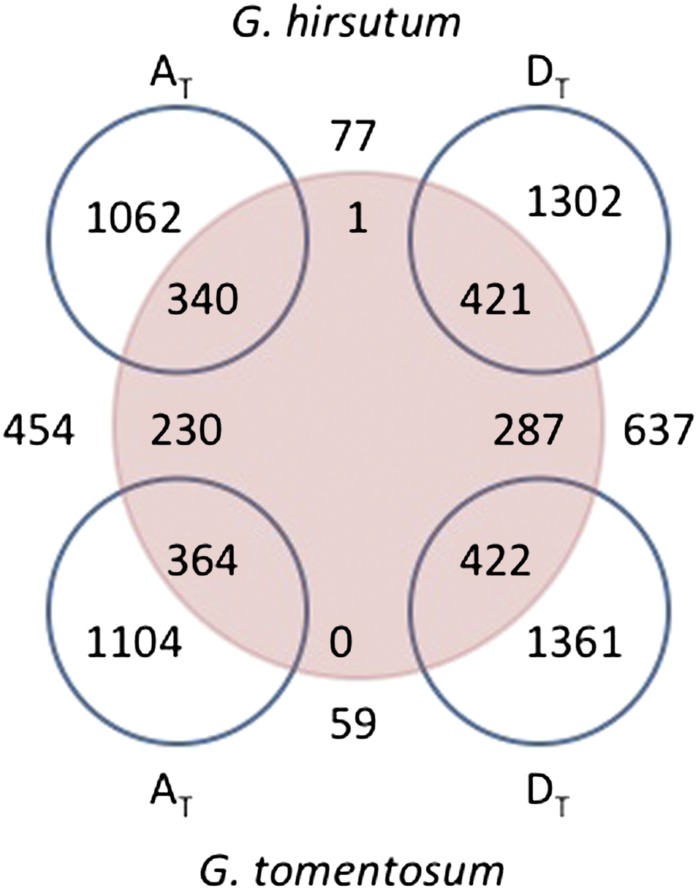
SNPs in *G. hirsutum* and *G. tomentosum* compared with the SNP index. Numbers inside blue circles represent the total number of SNPs for that genome, whereas underlined numbers between blue circles represent SNPs that are shared between two different allopolyploid genomes. This is not a formal Venn diagram because the numbers between blue circles are represented twice—once inside the circle(s) and once between the circles. They simply indicate the number of *shared* SNPs between the blue circles. Numbers inside the large red circle are indexed, while those outside were not contained within the SNP index.

After read categorization, SAMtools (Li *et al.* 2009) was used to call allele-SNPs within each genome-specific assembly (A_T_ and D_T_). These allele-SNPs represented loci that were heterozygous within the subgenomes of *G. hirsutum* and *G. tomentosum* (Figure 6). *G. tomentosum* had slightly more allele-SNPs, representing slightly more genes, than *G. hirsutum* (Table S3). The D_T_-genomes had more allele-SNPs, representing more genes, than their coresident A_T_-genomes. Approximately 75% of allele-SNPs were novel (*i.e.*, not indexed). A small number of indexed homoeo-SNPs also appeared as allele-SNPs within the genome-specific assemblies. These SNPs may reflect homeologous gene conversion events, or they may be false homoeo-SNPs.

By comparing the A_T_ and D_T_ alignments, we found that only a small number of novel homoeo-SNPs were identified in genic regions (77 in *G. hirsutum* and 59 in *G. tomentosum*), which suggests that most existing homoeo-SNPs between the A_T_- and D_T_- genomes were identified using the diploid genomes as surrogates. Therefore, increased sequencing of tetraploid transcriptomes will only minimally augment the number of “new” homoeo-SNPs; however, it would likely decrease the number of false-positive homoeo-SNPs resulting from diploid specific nucleotides.

## Discussion

### The phylogenetic context of SNPs

Read mapping in polyploid genomes is a natural application of DNA sequencing, although the practical challenges of mapping to the duplicated loci of polyploid genomes have not received much attention. These challenges include (1) mapping duplicated reads to a single reference genome, (2) the difference in similarity between the subgenomes of an allopolyploid and the diploid reference sequence, (3) gene conversion, (4) allopolyploid autapomorphies, and (5) diploid autapomorphies. Carefully classified SNPs can be used to address some of these challenges, despite the lack of a read-mapping program capable of mapping to a duplicated reference genome. For evolutionary and plant improvement studies, reads are best classified within a phylogenetic context using SNP positions and their corresponding nucleotides.

**Figure 7:**
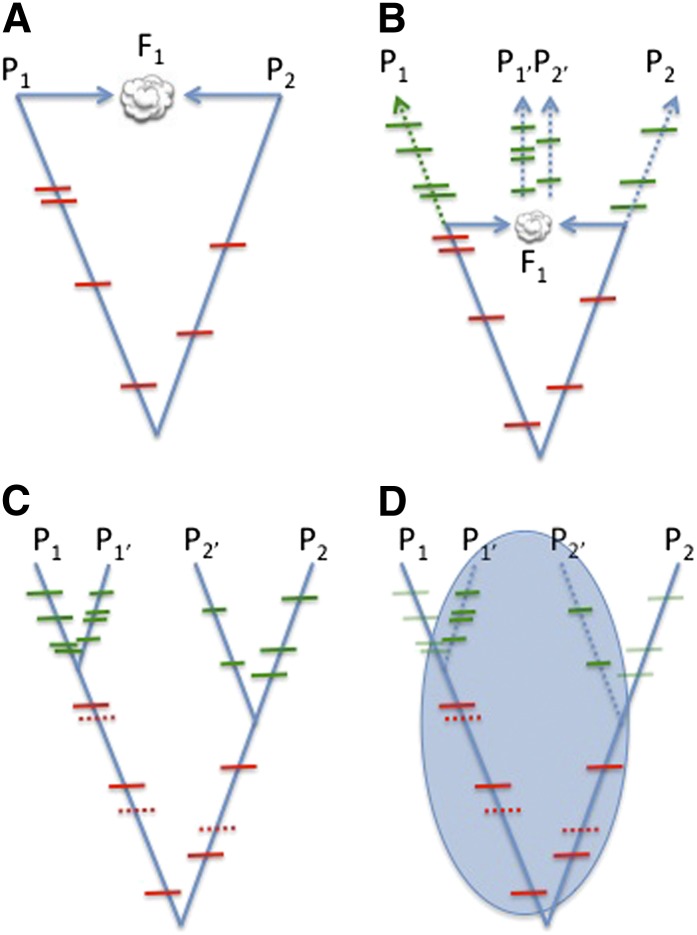
The phylogenetic contexts of SNPs within a polyploid genome. (A) Immediate formation of an F_1_ is largely additive in terms of DNA content. SNPs between the contributing diploid genomes can be readily detected in the newly formed hybrid (red SNP marks) and F_1_ reads can be readily categorized as originating from the P_1_ or P_2_ genome. (B) For most allopolyploids, a significant amount of time has passed since the initial genome duplication (represented by dashed arrows where time is on the y-axis). Nucleotide substitutions since polyploid formation (autapomorphies) resulted in allele-SNPs (green SNP marks). (C) SNPs can be placed within a classical phylogenetic context. Red and green SNP marks represent their respective SNP-types. Additional homoeo-SNPs (red, dashed SNP marks) were identified by comparing alignments of categorized reads (*e.g.*, A_T_-genome reference alignment to D_T_-genome reference alignment). (D) The blue circle represents identified SNPs (allele- and homoeo-SNPs) that are useful for improving mapping efficiencies of allopolyploid samples. Potential false-positive homoeo-SNPs (*i.e.*, diploid allele-SNPs) that are autapomorphic for each diploid do not negatively impact read mapping if one of the diploid alleles is common to one of the allopolyploid genomes.

In the simplest case involving allopolyploid formation, the genomes of Parent 1 (P_1_) and Parent 2 (P_2_) are combined into a common nucleus and form an F_1_ (Figure 7A). Assuming that such a sexually reproducing hybrid could be created, little nucleotide substitution will have occurred between the parental genomes and their counterparts within the polyploid F_1_ hybrid. Thus, SNPs between the diploid parents accurately predict homoeo-SNPs between the subgenomes of the F_1_, allowing for improvements in polyploid F_1_ read mapping efficiency and read categorization. For example, a sterile cotton diploid F_1_ hybrid (a nascent allopolyploid) was created by a recent hybridization. Categorization of reads from F_1_ had fewer chimeric (X) reads than reads from the natural allopolyploids (Figure 5).

This simple model of polyploidization lacks the passage of time since polyploid formation, during which additional nucleotide substitutions will have accumulated (autapomorphies in the diploid and polyploid genomes; Figure 7B). The nucleotide substitutions within each genome after polyploid formation are called allele-SNPs because (1) they occurred independently in various allopolyploid individuals (*e.g.*, accessions) and (2) they originated in only one genome and in only one of two germline chromosomes. After a single base substitution, drift, selection, or both will move the allele frequency of the derived base toward fixation or elimination. Thus, allele-SNPs can be found within individual genomes where a particular accession is heterozygous or by the comparison of two different homozygous accessions. These allele-SNPs would independently assort during meiosis after nucleotide substitution, regardless if they were identified in homozygous or heterozygous individuals. SNP identification efforts in other species have used confusing, alternative notation (*e.g.*, hemi-SNP, *etc*.) if the allele-SNPs were initially identified in a heterozygote as opposed to a homozygote (Bancroft *et al.* 2011; Harper *et al.* 2012). We do not use that context-dependent terminology in cotton.

Allele-SNPs can be identified by remapping categorized reads to the reference sequence and searching the alignments using common SNP-finding tools developed for diploid genomes (Li *et al.* 2009; McKenna *et al.* 2010). As an example, by using SAMtools we identified more than 1000 new allele-SNPs within both allopolyploid genomes of *G. hirsutum* and *G. tomentosum* (Figure 6). These allele-SNPs would be the most useful type of SNPs for cotton improvement because they have been bioinformatically discriminated from homoeo-SNPs and because they could be expected to segregate in Mendelian fashion (Van Deynze *et al.* 2009; Byers *et al.* 2012; Yu *et al.* 2012).

Comparison of independent alignments of categorized reads identified a limited number of new homoeo-SNPs because the extant diploid relatives used for initial homoeo-SNP identification were not perfect surrogates for the actual ancestral genomes that formed the ancestral allopolyploid, AND because of autapomorphic substitutions since polyploid formation (Figure 7). Resequencing multiple diploid accessions from each genome could identify the true diploid autopomorphies and reduce the number of SNPs erroneously classified as homoeo-SNPs. With our current dataset, these two SNP types were indistinguishable in our SNP index. Fortunately, the rate of false-positive homoeo-SNP (or false-positive allele-SNPs) had a negligible impact on read mapping because neither allele was penalized as a mismatch during SNP-tolerant read mapping. Thus, PolyCat used a conservative approach where if any SNP were included in the index (regardless of its source) its respective bases would be essentially masked during mapping.

Finally, SNPs can be placed on a traditional phylogenetic tree, but only a portion of those SNPs (homoeo-SNPs and allele-SNPs within the allopolyploid) impact mapping of sequence reads from allopolyploids (Figure 7). Allele-SNPs identified in subsequent re-sequencing of additional allopolyploid accessions can be easily added to the SNP index. Thus, improvement and extension of the PolyCat’s SNP index will be an iterative process (although SNP discovery will likely reach a saturation point and plateau). The combination of both types of SNPs (homoeo- and allelic) was included in the cotton SNP index for read mapping, and a similar collection of SNPs could be compiled for other allopolyploid genomes such as *Brassica napus* (Bancroft *et al.* 2011; Harper *et al.* 2012) and *Triticum aestivum* (Lai *et al.* 2012).

### Effectiveness of the PolyCat pipeline

The SNP index and read categorization process facilitated the analysis of allopolyploid cotton by reducing the bias in mapping efficiency between the two genomes and by providing a means to separate data generated for each allopolyploid genome (A_T_- and D_T_-genomes in cotton). Mapping all sequence reads to a single genome reference allowed for an aligned, comparative analysis between the two genomes within a given accession, as well as for more accurate analyses between accessions. Although these tools have been developed for cotton, they can be readily applied to any allopolyploid by providing an appropriate genome reference FASTA file, SNP index, and sequencing reads.

PolyCat is ultimately limited by the density of homoeo-SNPs across the genome. Reads belonging to a particular region of the genome can only be categorized if it has one or more homoeo-SNPs because every categorized read must overlap at least one SNP. The use of longer reads could improve the rate of categorization.

PolyCat is written in C++ and Perl, using BamTools (https://github.com/pezmaster31/bamtools) and Bioperl (Stajich *et al.* 2002). The custom scripts, the cotton SNP index, and a demo web application for demonstration of allopolyploid cotton read categorization are available online (http://bioinfo3.pgml.uga.edu/polyCat/upload.html). In the online version, 1 GB of sequence reads (non BS-seq) in FASTQ format can be categorized by PolyCat in approximately 15 min. Additional sequencing and development of software algorithms and tools will provide continued insights into polyploid genomes, their interactions, and their resultant phenotypes.

## Supplementary Material

Supporting Information
