# Supplementary material for: PolyCat: A Resource for Genome Categorization of Sequencing Reads From Allopolyploid Organisms
Source: G3 (Bethesda). 2013 Mar 1;3(3):517–25. doi: 10.1534/g3.112.005298 (PMC3583458; doi:10.1534/g3.112.005298)
Supplement: Supporting Information [file supp_3_3_517__index.html]

Supporting Information 

# PolyCat: A Resource for Genome Categorization of Sequencing Reads From Allopolyploid Organisms

## Supporting Information for Page, Gingle, and Udall, 2013

**Files in this Data Supplement:**

- Supporting Information - Tables S1-S3 (PDF, 148 KB)
- Table S1 - Transitions and Transversions in Cotton SNP index and Maize HapMaps 1 and 2 (PDF, 73 KB)
- Table S2 - Distribution of SNPs (homoeo- and allele-SNPs) across chromosomes (PDF, 67 KB)
- Table S3 - Heterozygous genes in *G. hirsutum* and *G. tomentosum* (PDF, 74 KB)
