# Supplementary material for: PolyCat: A Resource for Genome Categorization of Sequencing Reads From Allopolyploid Organisms
Source: G3 (Bethesda). 2013 Mar 1;3(3):517–25. doi: 10.1534/g3.112.005298 (PMC3583458; doi:10.1534/g3.112.005298)
Supplement: Supporting Information [file supp_3.3.517_005298SI.pdf]

G3 · Genes | Genomes | Genetics

PolyCat: A Resource for Genome Categorization of Sequencing Reads from Allopolyploid Organisms

Justin T. Page[*], Alan R. Gingle[§], and Joshua A. Udall[*]

* Plant and Wildlife Science Department, Brigham Young University, Provo, UT, 84062
§ Plant Genome Mapping Laboratory, University of Georgia, Athens, Georgia, 30602, USA

DOI: 10.1534/g3.112.005298

Table S1 Transitions and Transversions in Cotton SNP index and Maize HapMaps 1 and 2.

	**Cotton**		**Maize (gen 1)**		**Maize (gen 2)**	
C/T	770,845	29.7%	17,342,963	25.0%	16,980,248	33.5%
G/A	772,668	29.7%	10,604,324	25.6%	17,187,326	33.9%
**Transition**	**1,543,513**	**59.4%**	**27,947,287**	**50.6%**	**34,167,574**	**67.4%**
C/G	174,412	6.7%	3,177,765	9.2%	3,114,389	6.1%
A/T	381,403	14.7%	4,530,516	7.8%	4,430,958	8.7%
C/A	249,792	9.6%	4,630,754	15.4%	4,517,629	8.9%
T/G	249,872	9.6%	4,520,730	16.9%	4,436,275	8.8%
**Transversion**	**1,055,479**	**40.6%**	**16,859,765**	**49.4%**	**16,499,251**	**32.6%**
**Total**	**2,598,992**		**44,807,052**		**50,666,825**	

Table S2 Distribution of SNPs (homoeo- and allele-SNPs) across chromosomes.

Chromosome	Length (Mbp)	SNPs	SNPs/Kbp	% of total SNPs	Covered	Genic Length (Mbp)	Covered
Chr01	55.87	207,630	3.71	7.8	23.10%	8.72	61.00%
Chr02	62.77	198,479	3.16	7.5	20.00%	8.99	58.30%
Chr03	45.77	146,056	3.19	5.5	20.10%	6.06	59.50%
Chr04	62.18	215,057	3.45	8.1	21.50%	9.56	61.70%
Chr05	64.14	188,071	2.93	7.1	19.00%	8.87	58.30%
Chr06	51.07	218,317	4.27	8.2	25.30%	9.27	62.80%
Chr07	60.98	259,995	4.26	9.8	25.40%	12.18	63.10%
Chr08	57.13	216,505	3.78	8.2	23.50%	9.75	62.70%
Chr09	70.71	312,723	4.42	11.8	25.80%	14.87	63.60%
Chr10	62.18	182,100	2.92	6.9	18.60%	8.36	57.80%
Chr11	62.68	202,334	3.22	7.6	20.30%	9.59	58.70%
Chr12	35.43	120,941	3.41	4.5	21.50%	6.09	57.70%
Chr13	58.32	165,481	2.83	6.2	18.40%	8.64	58.40%

Table S3 Heterozygous genes in *G. hirsutum* and *G. tomentosum*.

	# Heterozygous Genes	Number of genes containing allele-SNPs in the SNP index	Number of genes containing allele-SNPs found within the polyploid alignments (not in index)
G. hirsutum-A_T	749	224	558
G. hirsutum-D_T	923	270	702
G. tomentosum-A_T	775	242	578
G. tomentosum-D_T	962	275	738
