# Supplementary material for: PolyCat: A Resource for Genome Categorization of Sequencing Reads From Allopolyploid Organisms
Source: G3 (Bethesda). 2013 Mar 1;3(3):517–25. doi: 10.1534/g3.112.005298 (PMC3583458; doi:10.1534/g3.112.005298)
Supplement: Supporting Information [file supp_3.3.517_TableS1.pdf]

Table S1 Transitions and Transversions in Cotton SNP index and Maize HapMaps 1 and 2.

	Cotton		Maize (gen 1)		Maize (gen 2)	
C/T	770,845	29.7%	17,342,963	25.0%	16,980,248	33.5%
G/A	772,668	29.7%	10,604,324	25.6%	17,187,326	33.9%
**Transition**	**1,543,513**	**59.4%**	**27,947,287**	**50.6%**	**34,167,574**	**67.4%**
C/G	174,412	6.7%	3,177,765	9.2%	3,114,389	6.1%
A/T	381,403	14.7%	4,530,516	7.8%	4,430,958	8.7%
C/A	249,792	9.6%	4,630,754	15.4%	4,517,629	8.9%
T/G	249,872	9.6%	4,520,730	16.9%	4,436,275	8.8%
**Transversion**	**1,055,479**	**40.6%**	**16,859,765**	**49.4%**	**16,499,251**	**32.6%**
**Total**	**2,598,992**		**44,807,052**		**50,666,825**	
