# Supplementary material for: PolyCat: A Resource for Genome Categorization of Sequencing Reads From Allopolyploid Organisms
Source: G3 (Bethesda). 2013 Mar 1;3(3):517–25. doi: 10.1534/g3.112.005298 (PMC3583458; doi:10.1534/g3.112.005298)
Supplement: Supporting Information [file supp_3.3.517_TableS2.pdf]

Table S2 Distribution of SNPs (homoeo- and allele-SNPs) across chromosomes.

Chromosome	Length (Mbp)	SNPs	SNPs/Kbp	% of total SNPs	Covered	Genic Length (Mbp)	Covered
Chr01	55.87	207,630	3.71	7.8	23.10%	8.72	61.00%
Chr02	62.77	198,479	3.16	7.5	20.00%	8.99	58.30%
Chr03	45.77	146,056	3.19	5.5	20.10%	6.06	59.50%
Chr04	62.18	215,057	3.45	8.1	21.50%	9.56	61.70%
Chr05	64.14	188,071	2.93	7.1	19.00%	8.87	58.30%
Chr06	51.07	218,317	4.27	8.2	25.30%	9.27	62.80%
Chr07	60.98	259,995	4.26	9.8	25.40%	12.18	63.10%
Chr08	57.13	216,505	3.78	8.2	23.50%	9.75	62.70%
Chr09	70.71	312,723	4.42	11.8	25.80%	14.87	63.60%
Chr10	62.18	182,100	2.92	6.9	18.60%	8.36	57.80%
Chr11	62.68	202,334	3.22	7.6	20.30%	9.59	58.70%
Chr12	35.43	120,941	3.41	4.5	21.50%	6.09	57.70%
Chr13	58.32	165,481	2.83	6.2	18.40%	8.64	58.40%
