# Supplementary material for: PolyCat: A Resource for Genome Categorization of Sequencing Reads From Allopolyploid Organisms
Source: G3 (Bethesda). 2013 Mar 1;3(3):517–25. doi: 10.1534/g3.112.005298 (PMC3583458; doi:10.1534/g3.112.005298)
Supplement: Supporting Information [file supp_3.3.517_TableS3.pdf]

Table S3 Heterozygous genes in *G. hirsutum* and *G. tomentosum*.

	# Heterozygous Genes	Number of genes containing allele-SNPs in the SNP index	Number of genes containing allele-SNPs found within the polyploid alignments (not in index)
G. hirsutum-A_T	749	224	558
G. hirsutum-D_T	923	270	702
G. tomentosum-A_T	775	242	578
G. tomentosum-D_T	962	275	738
